# Multimodal EEG, ECG, and video dataset of yoga practitioners during concentration and mind-wandering tasks

**DOI:** 10.1038/s41597-026-07209-z

**Published:** 2026-04-10

**Authors:** Alexey Kashevnik, Eduard Glekler, Elena Artemenko, Ivan Brak, Irina Shoshina, Vladimir Romaniuk

**Affiliations:** 1 SPC RAS, 14 Line V.O. 39, 199178 St. Petersburg, Russia; 2 HSE University, Social and Cognitive Informatics Lab, Sedova st. 55/2, St. Petersburg, 192148 Russia; 3 MISIS University of Science and Technology, Leninsky pr. 4-1, 119049 Moscow, Russia; 4 RUDN University, Miklukho-Maklaya str.6, 117198 Moscow, Russia

**Keywords:** Neurophysiology, Consciousness

## Abstract

We present a multimodal dataset containing electroencephalography (EEG), electrocardiography (ECG), and video recordings from 49 participants. Each participant completed a single session lasting approximately 45 minutes. During each session they performed five tasks: resting state, inward concentration (focusing on the center of the forehead), outward concentration (visual search), and mind-wandering. EEG data were recorded from 64 channels at 2048 Hz; ECG and frontal video were collected simultaneously. The dataset includes 49 validated recordings: 25 from experienced yoga practitioners and 24 from individuals without prior experience in yoga or other self-regulation practices. The recordings are synchronized and structured for convenient use in cross-modal analyses. This dataset may support research in attention, concentration, meditation, affective computing, and multimodal signal integration. All data are anonymized and organized for easy access and reuse.

## Background & Summary

Analysis of electroencephalogram (EEG) data is one of the most widely used methods for recognizing and assessing human functional states. EEG signals are utilized for this purpose both in scientific research and in the operation of personal devices such as Muse^1^, Emotiv^2^, and others. These devices are commonly used in self-regulation practices, including concentration and meditation, to assess their quality and progress. However, based on an analysis of scientific literature on recognizing and assessing concentration states using EEG signals, the authors have not identified a unified set of metrics applicable for these purposes. This may be due to weak correlation of these metrics with concentration states or differences in the definition of the term “concentration” itself.

For example, in^3^, three states are analyzed: relaxation (without concentration), concentration (focus on a point), and immersion (focus on a video game). The immersion state, which requires quick reactions and sustained attention, is conceptually closer to the idea of concentration used in most other studies than the state referred to as “concentration” in this work (a similar state is mentioned only in^4^). Nevertheless, both of these states significantly differ from the relaxed baseline state, as evidenced by changes in signal power in the *θ*, *α*, and *β* bands. The *θ*/*α* ratio emerged as the most universal indicator for distinguishing these states, while *β*/*θ* did not show consistent changes, contradicting findings from [5], where *β*/*θ* indicated changes associated with concentration.

Studies^5^ and^6^ explore the impact of environmental conditions (temperature and lighting, respectively) on the learning process using the metric (*SMR* + *Middleβ*)/*θ*. Despite differing experimental conditions, both studies observed a significant influence of concentration on this metric. A similar metric, measured exclusively from prefrontal electrodes, was used in^7^ and^8^, where in^7^, signals were also recorded from other sites, but only prefrontal data were used for metric computation. Notably, that both studies included only male participants to eliminate potential influences of the menstrual cycle.

Other studies, such as^9^ and^8^, included female participants and focusing on more formalized concentration tasks such as text proofreading and performing cognitive tests under different lighting conditions. These studies stand out by using additional objective control metrics, such as cortisol levels and task performance accuracy. In^9^, the significance of the *θ*/*β* metric is highlighted, which is largely the inverse of (*SMR* + *Middleβ*)/*θ*. However, in^8^, no significant correlation was observed for power in the *θ* band, although correlations were present in the *δ*, *α*, *β*_1_, and *β*_2_ bands.

From this literature analysis, it is evident that spectral analysis, including the computation of power in the *θ*, *α*, and *β* bands and their ratios, is the primary method for recognizing concentration states. Nevertheless, studies^4^ and^10^ criticize this approach, pointing out low accuracy due to the nonlinear and non-stationary nature of EEG signals and suggesting alternative non-spectral metrics (particularly entropy-based metrics, which proved more accurate for concentration recognition). In^10^, non-spectral indicators such as OCN and MSFEn yielded comparable results even when calculated from a single prefrontal lead instead of 63.

Studies including^11^^–^^14^ investigate the state of mindfulness meditation, which is similar to the state of internal concentration in yoga. Although their methodologies differ (primarily comparing experienced meditators with novices), many of the same metrics (both spectral and non-spectral) are employed. Overall, spectral metrics are predominant, and in^11^, an LDS analysis demonstrated interdependence between spectral metrics and subjective psychological measures (YBOCS, FFMQ), highlighting their relevance.

Therefore, spectral metrics, such as (*SMR* + *Middleβ*)/*θ*, are widely used for concentration assessment. At the same time, alternative entropy-based methods are gaining attention due to higher accuracy. Cross-study comparisons are complicated by differing definitions of concentration, variations in baseline conditions, and experimental setups Nevertheless, most studies show significant EEG signal changes associated with concentration, and a minimal set of prefrontal leads often suffices for analysis.

Importantly, EEG analysis is not the only method for assessing concentration states. Various devices, such as InnerBalance^15^, use electrocardiogram (ECG) analysis for similar purposes. Compared to EEG, devices for recording ECG in acceptable quality are significantly more accessible for everyday use at home.

In addition, the authors’ own research indicates that video recordings during concentration can provide physiological parameters such as respiratory rate^16^, heart rate^17^, and blood oxygen saturation^18^. Detecting correlations between these parameters and concentration states could reduce the need for specialized hardware, enabling mobile-phone-only solutions.

Finally, combining EEG, ECG, and video data allows mutual cross-validation between modalities - an essential step given the lack of objective, standalone metrics for concentration. Consequently, future research in this area should prioritize parallel EEG, ECG, and video recordings across both concentration and baseline states, involving both experienced practitioners and control groups.

## Methods

### Participants

**Fig. 1:**
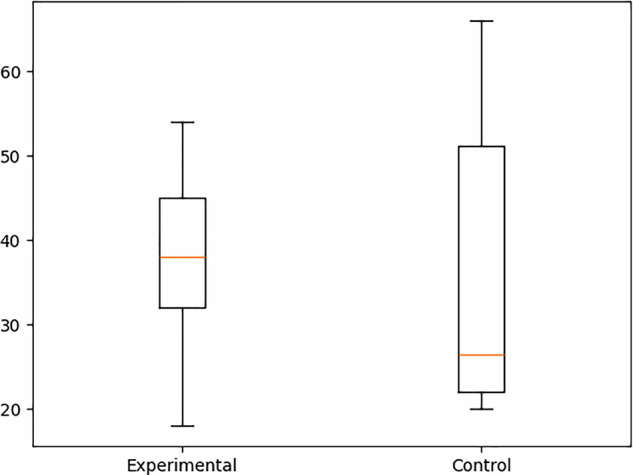
Age distribution of experimental and control groups.

The study involved 33 participants (Table 1), including 15 individuals with more than one year of experience in yoga and meditation practices (37 ± 10 years old, 11 female, see Fig. 1). The remaining 16 participants, with no prior experience in self-regulation practices, served as the control group (36 ± 17 years old, 10 female, see Fig. 1). Although there are significant age differences between the participant groups, this study does not focus on group differences but rather on the differences between functional states for each individual participant. The extent of these differences is assumed to depend significantly more on experience rather than on the participant’s age. All participants were informed about the study procedures and provided written informed consent. They were also notified of the possibility of open publication of their data in anonymized form, with no personally identifiable information included. Participants self-reported their experience with body-focused concentration practices and confirmed the absence of any diagnosed mental health conditions. All published data were fully anonymized to ensure participant confidentiality.

**Table 1:** Description of the participants (Rec Id - identifier of recordings, Sub Id - participant id, Control - flag that shows is the participant from the experimental or from control group, gender - participant gender, age - participant age, Experience - show the experienct in yoga practices in years, Sleep - number of hours that participant sleep last night, CN1.CN4 - subjective estimation of concentration efficiency of the participant during the concentration task by quartiles).

Sub Id	Session	ECG	Video	Control	Gender	Age	Experience	Sleep	CN1	CN2	CN3	CN4
sub-001	1	0	0	0	F	50	20	7	0	0	0	0
sub-002	1	1	1	0	F	38	7	7	8	7	4	7
sub-002	2	1	1	0	F	38	7	7	8	6	6	8
sub-002	3	1	1	0	F	38	7	7	0	0	0	0
sub-003	1	1	1	0	M	42	7	7	0	0	0	0
sub-003	2	1	1	0	M	42	7	6	8	5	6	8
sub-003	3	1	1	0	M	42	7	6.5	8	8	8	6
sub-004	1	1	1	0	M	49	30	4	7	5	8	5
sub-005	1	1	1	0	M	32	5	6	8	8	8	8
sub-005	2	1	1	0	M	32	5	5	8	8	8	8
sub-006	1	1	1	0	F	20	3	6	3	6	10	8
sub-007	1	1	1	0	F	30	3.5	8	6	8	6	9
sub-008	1	1	1	1	F	38	1	7	7	7	6	7
sub-009	1	1	1	0	F	53	9	7	7	5	2	1
sub-009	2	1	1	0	F	53	9	9	6	8	9	9
sub-009	3	1	1	0	F	53	9	8	2	2	2	3
sub-010	1	1	1	0	F	49	25	5.5	3	3	3	3
sub-011	1	1	1	0	F	42	5	8	4	5	6	9
sub-012	1	1	1	0	F	33	5	5	7	6	5	5
sub-013	1	1	1	1	F	18	2	8	8	6	5	8
sub-014	1	1	1	0	M	33	3.5	8	6	7	7	8
sub-015	1	1	1	0	M	40	5	4	6	5	7	8
sub-016	1	1	1	1	M	41	0	6	6	5	4	5
sub-017	1	1	0	1	M	25	0	6	1	1	1	2
sub-018	1	1	0	1	F	33	0	11	3	4	3	2
sub-019	1	1	1	1	F	22	0	6	3	8	8	5
sub-020	1	1	1	1	F	22	0	7	8	6	5	6
sub-021	1	1	1	0	F	22	0	7	10	8	6	9
sub-022	1	1	1	1	M	21	0	7	3	8	5	8
sub-023	1	1	1	1	F	22	0	6	7	3	5	6
sub-024	1	1	1	1	M	21	0	8.5	7	8	6	8
sub-025	1	1	1	1	M	28	0	8	10	7	8	10
sub-025	2	1	1	1	M	28	0	8	9	7	7	7
sub-025	3	1	1	1	M	28	0	8	8	6	5	7
sub-026	1	1	1	1	F	20	0	12	7	4	6	5
sub-027	1	1	1	1	F	51	0	7	7	5	5	7
sub-027	2	1	1	1	F	51	0	7	8	9	8	7
sub-027	3	1	1	1	F	51	0	6	3	3	7	7
sub-028	1	1	1	1	M	63	0	7	6	5	3	2
sub-029	1	1	1	1	F	51	0	6	8	3	6	2
sub-029	2	1	1	1	F	51	0	6	5	5	5	7
sub-029	3	1	1	1	F	51	0	7	7	5	7	5
sub-030	1	1	1	1	F	66	0	7	8	4	7	5
sub-031	1	1	1	1	F	64	0	6	7	3	5	2
sub-031	2	1	1	1	F	64	0	7	8	7	7	1
sub-031	3	1	1	1	F	64	0	7	6	5	4	1
sub-032	1	1	1	1	M	24	1	7	4	8	4	9
sub-033	1	1	1	0	F	45	4	7	10	6	8	9
sub-033	2	1	1	0	F	45	4	7	10	8	8	9

### Environment, equipment, and procedure

All participants completed the experiment using a monitor connected to a workstation equipped with an Intel Core i7 processor and running Windows 11. The presentation screen resolution was 2560 × 1440 pixels. Participants were seated comfortably in a chair at a distance of 65-70 cm from the display monitor. The distance was adjusted individually, with the constraint that the webcam (ASUS Webcam C3) mounted on the monitor had to fully capture the participant’s face and chest.

Stimuli were presented using the EventIDE software (OkazoLab Ltd.) via real-time data streaming. Task instructions were delivered automatically, and synchronized event markers were transmitted from EventIDE to the EEG recording system via the Lab Streaming Layer (LSL) protocol. The EEG system consisted of an eego mylab (EE-225) bioamplifier (ANT Neuro) connected to a tablet running the eego software. The workstation and tablet operated within the same local network to ensure synchronized data exchange.

EEG signals were recorded continuously at a sampling rate of 2048 Hz using 64 Ag/AgCl electrodes placed according to the international 10-10 system, with the ground electrode at GND and the reference at CPz. Electrode impedance did not exceed 10 *k*Ω. ECG data were collected in a bipolar configuration using dry electrodes attached to the participant’s wrists.

**Fig. 2:**
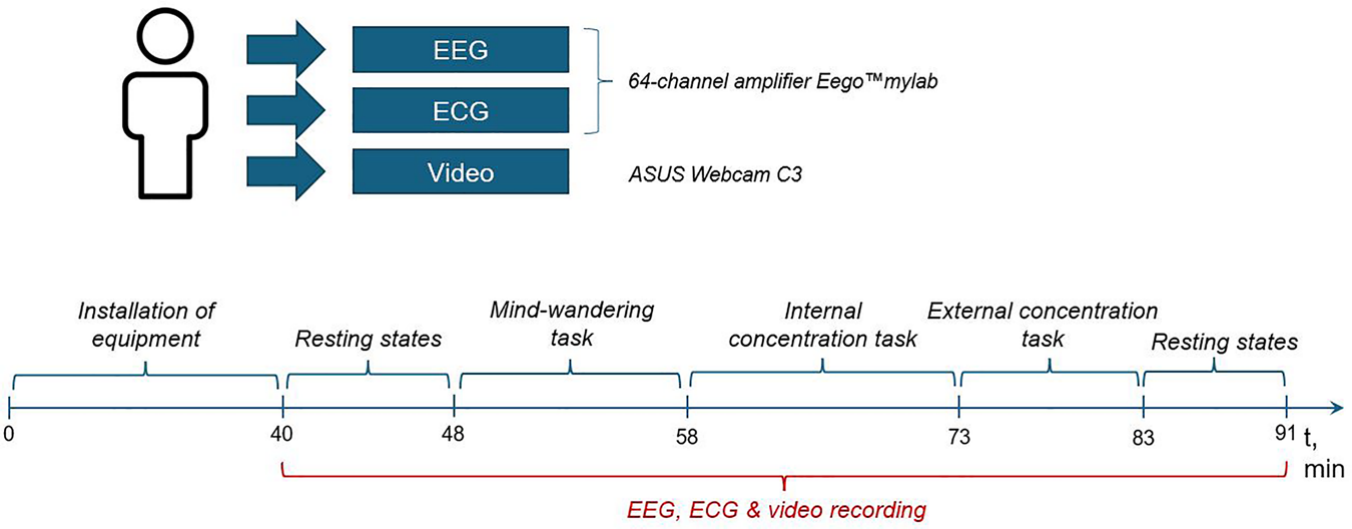
Proposed methodology for data acquisition.

Simultaneously, video recordings were captured using the Windows Camera application at a resolution of 1920 × 1080 pixels and a frame rate of 30 frames per second (see Fig. 2). The room was not subject to strict control over temperature, lighting, or ambient noise, but conditions remained within comfortable limits. None of the participants reported any discomfort or environmental interference with task performance.

Each experimental session lasted approximately 45 minutes and included five tasks performed consecutively without breaks. Tasks 1 and 5 (at the beginning and end of the session) involved resting states with eyes alternately open and closed. Tasks 2, 3, and 4 required the participant to enter states of free mind-wandering, internal concentration (focusing on the center of the forehead), and external concentration (visual search), respectively.

After completing the session, participants filled out a survey assessing their current state and the level of concentration experienced during each task.

#### Task 1: Resting states

For Task 1, participants were given four instructions, each occurring at two-minute intervals: “Open your eyes,” “Close your eyes,” “Open your eyes,” “Close your eyes.” Additionally, for this task and all subsequent ones, participants were given a general instruction to remain as still as possible, avoid frequent blinking, and refrain from rotating their eyes.

#### Task 2: Mind-wandering task

Task 2 involved recording the background state, opposite to a state of concentration, namely, a state of mind-wandering. Participants were instructed to sit still with their eyes closed and to allow their thoughts to flow freely from one to another, while trying not to fall asleep. The duration of this task was 10 minutes, after which participants immediately proceeded to the next task without any break.

#### Task 3: Internal concentration task

Task 3 required participants to engage in internal concentration by focusing on an imagined point. They were instructed to sit still with their eyes closed and direct their attention to the center of their forehead. If they became distracted, they were asked to gently redirect their attention back to that point. The task lasted for 15 minutes.

#### Task 4: External concentration task

For Task 4, participants performed a complex visual search task that required identifying a target character within densely populated and visually rich scenes. These images, sourced from publicly available online materials, were selected to resemble classic “find-the-character” puzzles, with multiple distractors surrounding a single target. At the beginning of the task, participants were shown a reference image of the target character, whom they were asked to locate in each subsequent image. Upon visually identifying the target, participants confirmed their discovery verbally, after which a new image was presented. The task continued until the participant successfully located the target in four different images or until 10 minutes had elapsed, whichever occurred first.

#### Task 5: Resting states

For Task 5 the same as for the Task 1, participants were given four instructions, each occurring at two-minute intervals: “Open your eyes,” “Close your eyes,” “Open your eyes,” “Close your eyes”.

#### Survey

After each session, participants completed a survey in which they were asked to provide information about their experience with concentration practices and the number of hours of sleep the previous night. They were also asked to subjectively assess the quality of their concentration by mentally dividing it into quarters and separately rating the level of concentration within each quarter on a ten-point scale, where 1 indicated a complete lack of concentration, and 10 represented the best concentration they had ever experienced.

### Ethical Approval

St. Petersburg Federal Research Center of the Russian Academy of Sciences ethics committee provided ethical approval for the acquiring the experiments and data capturing, including confirmation the that informed consent for participation and data sharing was obtained from each participant (protocol 7 from 26.06.2025).

## Data Records

The dataset is available at^19^ followed by a practical description of the files, including file names and folder structure.

### EEG data records

**Fig. 3:**
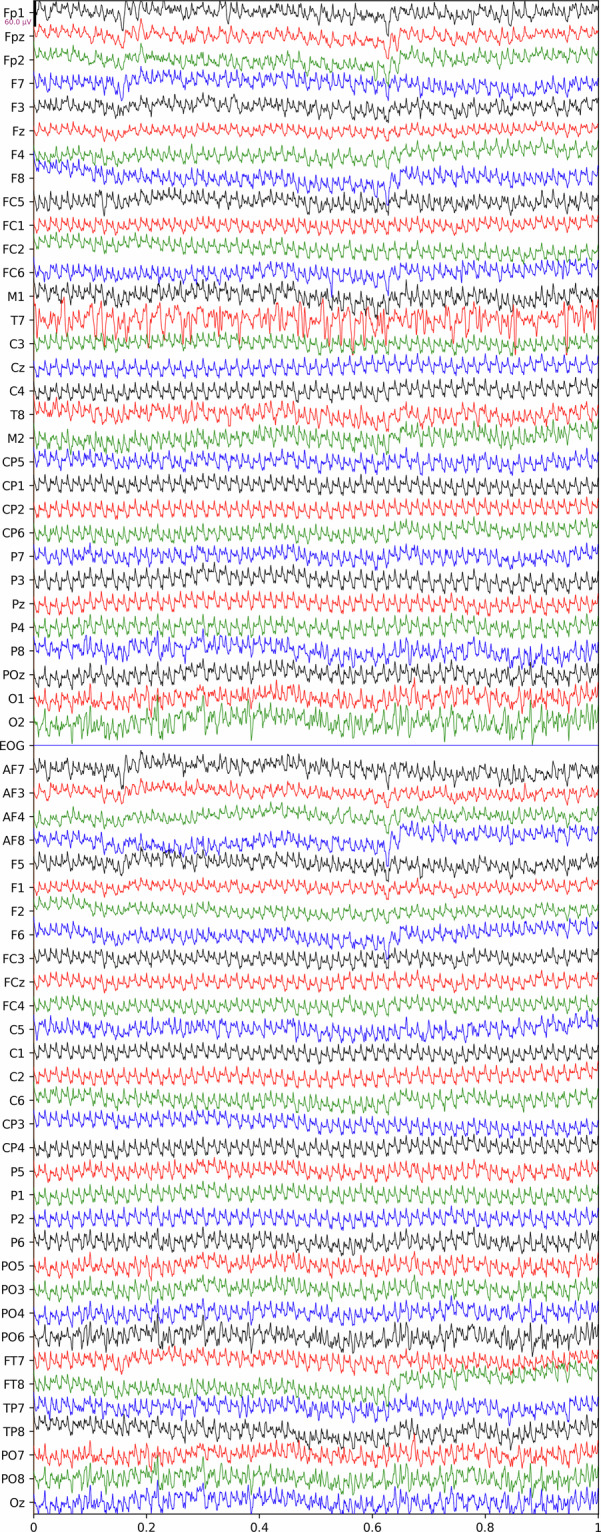
Sample EEG segment.

For each subject and session, EEG recordings are stored in the directory *sub* − *XXX*/*ses* − *YY*/*eeg* using the BrainVision file format, consisting of three files:.vhdr,.vmrk, and.eeg. The.vhdr file contains meta-information about the record and references to the.vmrk and.eeg files for reading the BrainVision format in various applications. When loading the data in compatible applications, the.vhdr file should be specified as the entry point. The.vmrk file contains information about events with corresponding timestamps. In this case, it stores the timestamps for the start and end of tasks performed by the participant. The.eeg file contains the actual signal data from various EEG channels. File naming follows the BIDS convention: *sub* − *XXX*_*ses* − *YY*_*task* − *default*_*run* − 01_*eeg*, where the subject, session, task label, and run index of the recording are specified, respectively. Recording-level metadata is provided in the additional file _*e*_*eg*. *json*. Information about channel names and types is given in _*c*_*hannels*. *tsv*. Event annotations with their timing are stored in _*e*_*vents*. *tsv*. To determine the positions of the EEG electrodes, the standard 10-10 montage should be used. All channels in records have names that can be mapped to the corresponding electrode positions. Figure 3 shows a one second ECG recording snapshot of data.

### ECG data records

**Fig. 4:**
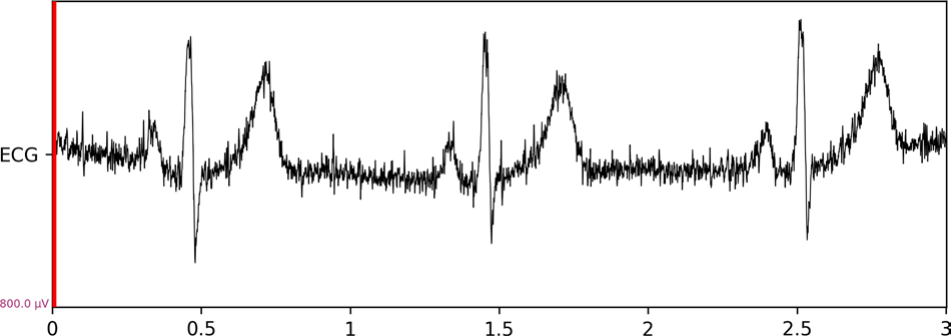
Sample ECG segment.

ECG data are stored together with EEG data in the corresponding files. A separate channel with the appropriate type is allocated for storing this data. The ECG data can be extracted like any other channel data as a time series, after which they can undergo any desired processing. Figure 4 shows a three seconds of ECG recording snapshot.

### Video records

**Fig. 5:**
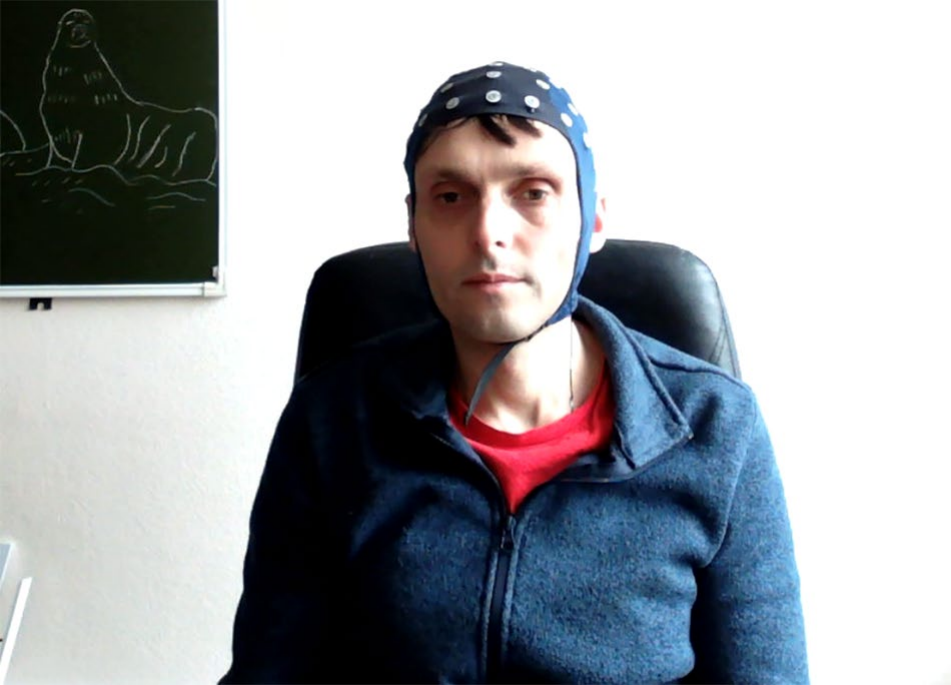
Video frame sample.

Video recordings, when available, are stored within the session-specific behavioural directory *sub* − *XXX*/*ses* − *YY*/*beh* as MP4 files and accompanied by a JSON file that describes recording metadata. The video and EEG recordings can be aligned based on the moments of eye opening/closing and the corresponding event markers in the EEG data. For some EEG recordings, there are no corresponding video files due to their non-compliance with the criteria specified earlier. The sample frame is shown on Fig. 5.

### Data folders

**Fig. 6:**
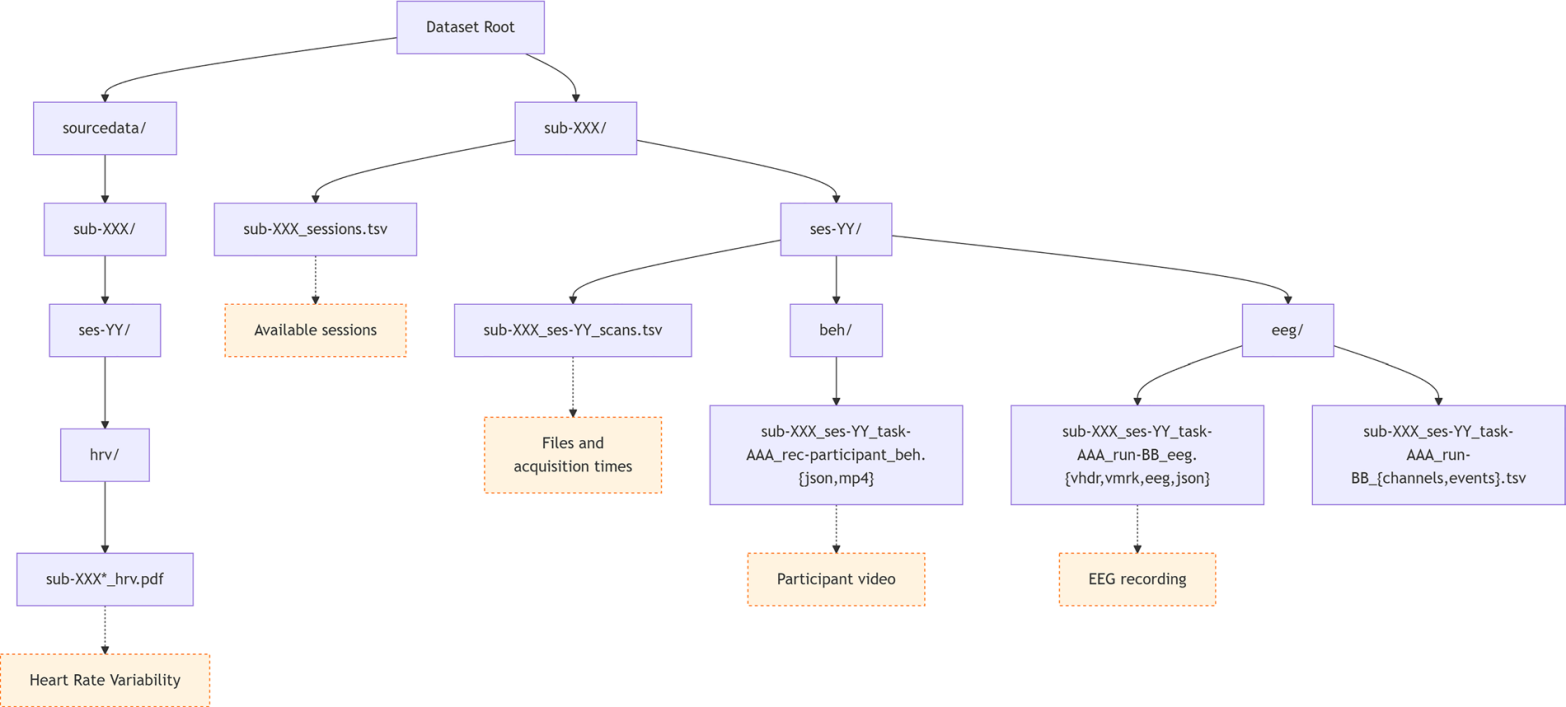
Dataset folder structure.

Dataset is organised according to the Brain Imaging Data Structure specification for electrophysiology (See Fig. 6). At the dataset root level, general metadata are provided in *dataset*_*description*. *json*, while participant-level information is stored in *participants*. *tsv*, including the participant’s Id, control group affiliation, age, concentration experience, and whether ECG and video recordings are present or absent. The primary data are arranged into subject directories named *sub* − *XXX*, and divided by session directories named *ses* − *YY*. Materials that are not part of the BIDS raw data hierarchy are stored under *sourcedata* and use the same subject and session identifiers.

At the subject level, each *sub* − *XXX* directory contains *sub* − *XXX*_*sessions*. *tsv*, which enumerates available sessions, hours of sleep the night before the recording, the participant’s self-assessment of concentration quality by quarters (a dash indicates that the participant could not provide an assessment). Each session directory contains *sub* − *XXX*_*ses* − *YY*_*scans*. *tsv*, which lists files for that session. Within each session, electrophysiological recordings are placed in *eeg* and behavioral video recordings are placed in *beh*.

## Technical Validation

### EEG data validation

To validate EEG data and its relevance for recognizing concentration based on the studies reviewed earlier, the following set of metrics was identified as potentially applicable for detecting this state:

- Signal power in the *α*-band
- Signal power in the lower *β*-band (up to 20 Hz)
- Signal power in the *θ*-band
- Signal power in the SMR-band
- Signal entropy in the range from 0.3 to 30 Hz
- Signal entropy in the *α*-band
- Signal entropy in the lower *β*-band (up to 20 Hz)
- Signal entropy in the *θ*-band
- Signal entropy in the SMR-band

The point-biserial correlation coefficient (PBCC) was used as an evaluation metric to determine the applicability of these metrics for recognition. Additionally, due to the variability of the processes under consideration, an analogous coefficient, PBCCIQR, was calculated based on interquartile range values of the metrics rather than their mean values^20^, using formula (1).

1 $$PBCCIQR=\frac{IQ{R}_{1}-IQ{R}_{0}}{{s}_{n-1}}\sqrt{\frac{{n}_{1}{n}_{0}}{n(n-1)}}$$

All metrics were calculated both as averages across all channels and for subsets of channels corresponding to different brain regions. This approach was informed by previous studies, which demonstrated variability in the applicability of signals from different regions (in particular, most studies noted the high relevance of prefrontal channels).

The following channel groups were selected for consideration^21^:

- Global: all 63 electrodes;
- Prefrontal: F3, Fz, F4;
- Central: C3, Cz, C4;
- Parietal and Occipital: O1, O2, P3, P4.

Since no artifact removal methods are applied in this work, the prefrontal channels Fp1 and Fp2, commonly used in other studies, were replaced by less artifact-prone channels: F3, Fz, F4. For the calculation of spectral metrics, standard tools from the MNE library were used. After filtering the signal with an FIR filter, it was segmented into 2-second non-overlapping epochs. Signal power in the relevant frequency bands was then computed for each epoch using Welch’s method. Entropy metrics were computed using tools from the MNE_features library after additional signal filtering with a similar FIR filter in the relevant frequency range^22^. Sample entropy (SampEn)^23^, calculated using formula (2), was used as the entropy metric.

2 $$A(m,r)=\mathop{\sum }\limits_{i=1}^{N-m+1}C(i,r,m)$$

3 $$SampEn(m,r)=-\log \left(\frac{A(m,r)}{A(m+1,r)}\right)$$

After calculating the metrics for each channel, their values were averaged across the channels in the corresponding region. Metric significance was evaluated using PBCC and PBCCIQR for the resulting values within two states (specifically, mind-wandering and concentration), excluding the lowest and highest 2.5% of values to partially mitigate the impact of artifacts without manual preprocessing of the signal.

**Fig. 7:**
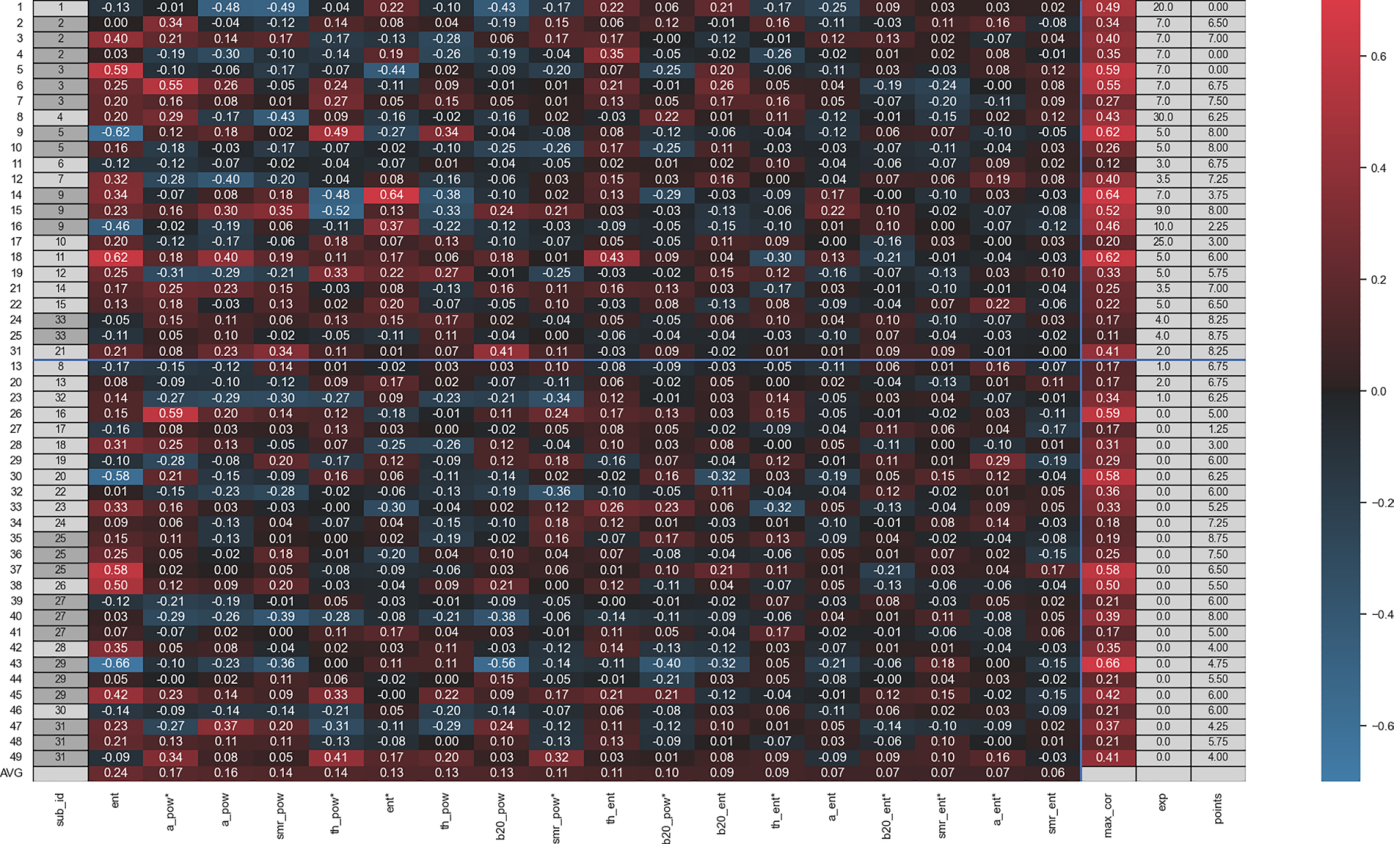
Global matrix (for all 63 electrodes) that shows difference (PBCC) between concentration and mind wandering for every considered metrics. Metrics with * show difference by interquartile range (PBCCIQR). Columns are sorted based on metrics significance.

The results of the metric significance evaluation are presented in Fig. 7 as a matrix of correlation coefficient values. Columns are sorted in descending order of the average absolute values of the metrics, indicated in the AVG row. Rows are sorted by participants (with recordings from the same participant grouped consecutively). The recording index is indicated in the rec_id column. The exp, points, and max_cor columns show participant experience, the average subjective assessment of concentration quality for the second and third quarters, and the maximum correlation coefficient among all metrics, respectively. A value of 0 in the points column means the participant could not assess concentration quality, while -1 indicates that instructions were not followed correctly. The other columns contain correlation coefficient (PBCC) values for entropy and power metrics in the *α*, *β* (up to 20 Hz), *θ*, and SMR bands. PBCCIQR values are marked with an asterisk (^*^). For example, the b20_ent column corresponds to PBCCIQR for entropy in the *β*-band, while smr_pow corresponds to PBCC for SMR power.

It can be observed that more experienced participants exhibit significant differences between states for at least one metric. This does not reveal a general concentration state pattern but confirms the distinctness of these states for each specific recording. The average value of max_cor for the experimental group is 0.38. For the control group, it is 0.33. Thus, significant differences are observed between the groups, indicating that the state of concentration is more distinguishable in the experimental group.

**Fig. 8:**
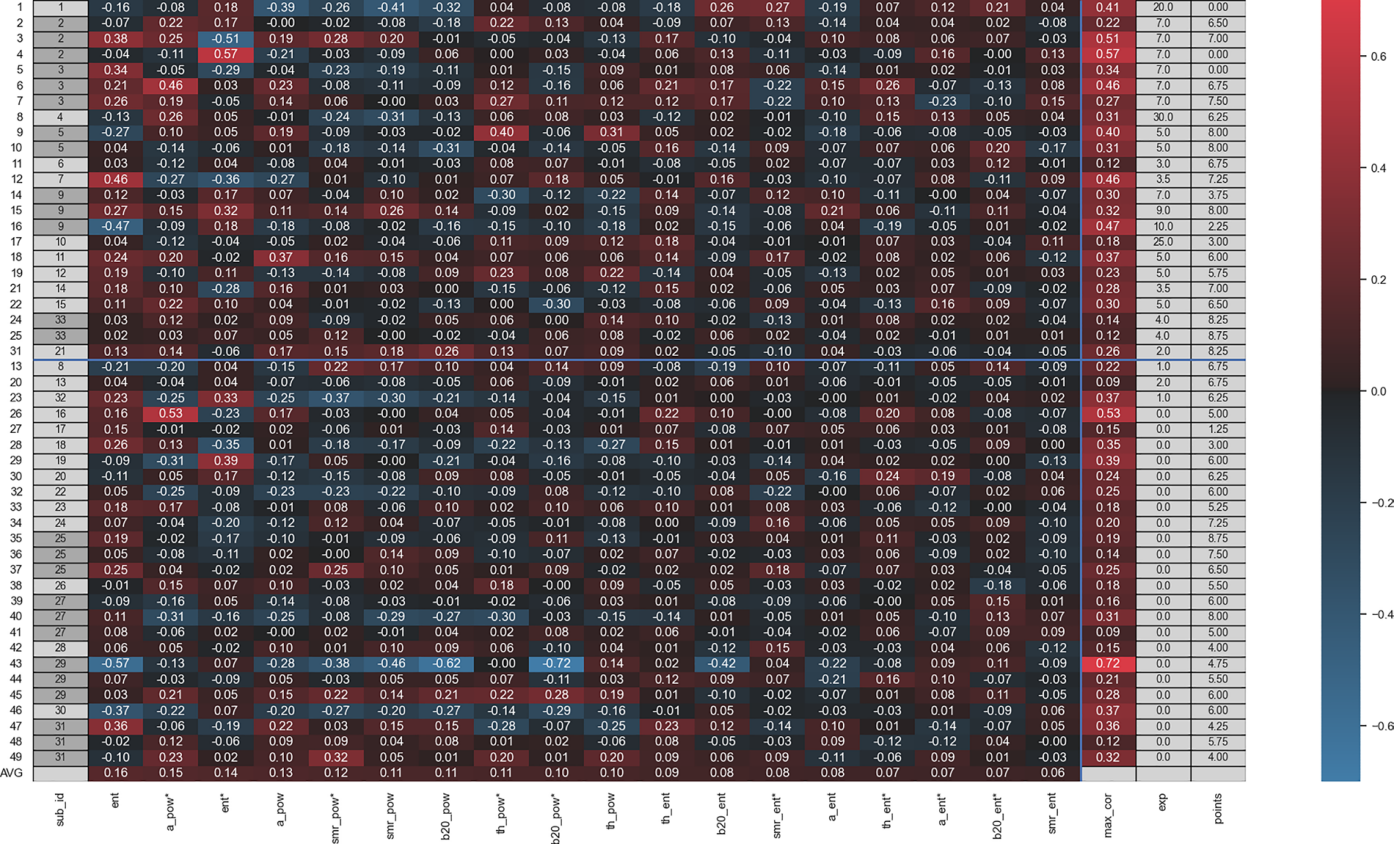
Frontal matrix (for frontal electrodes) that shows difference (PBCC) between concentration and mind wandering for every considered metrics. Metrics with * show difference by interquartile range (PBCCIQR). Columns are sorted based on metrics significance.

**Fig. 9:**
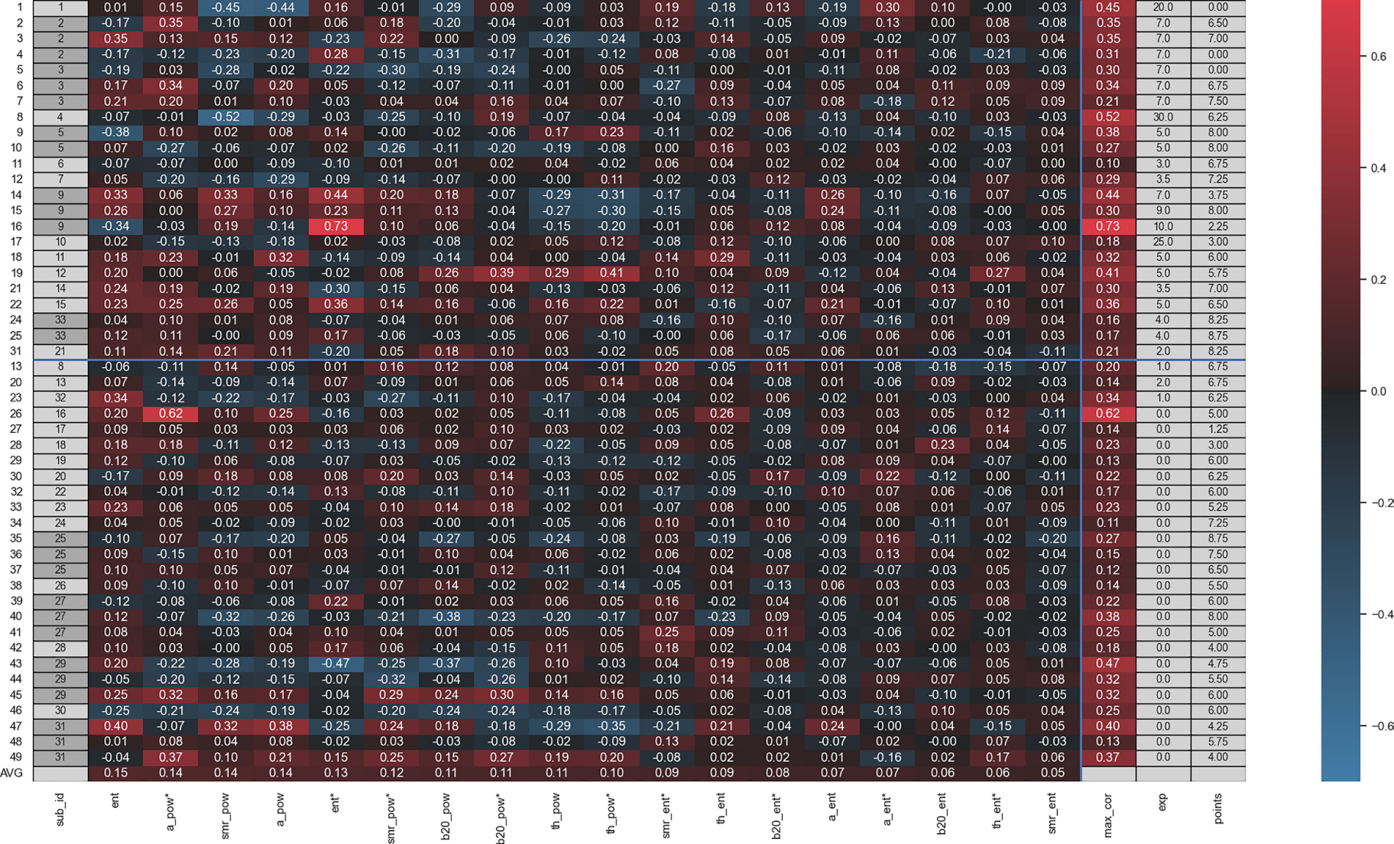
Central matrix (for central electrodes) that shows difference (PBCC) between concentration and mind wandering for every considered metrics. Metrics with * show difference by interquartile range (PBCCIQR). Columns are sorted based on metrics significance.

**Fig. 10:**
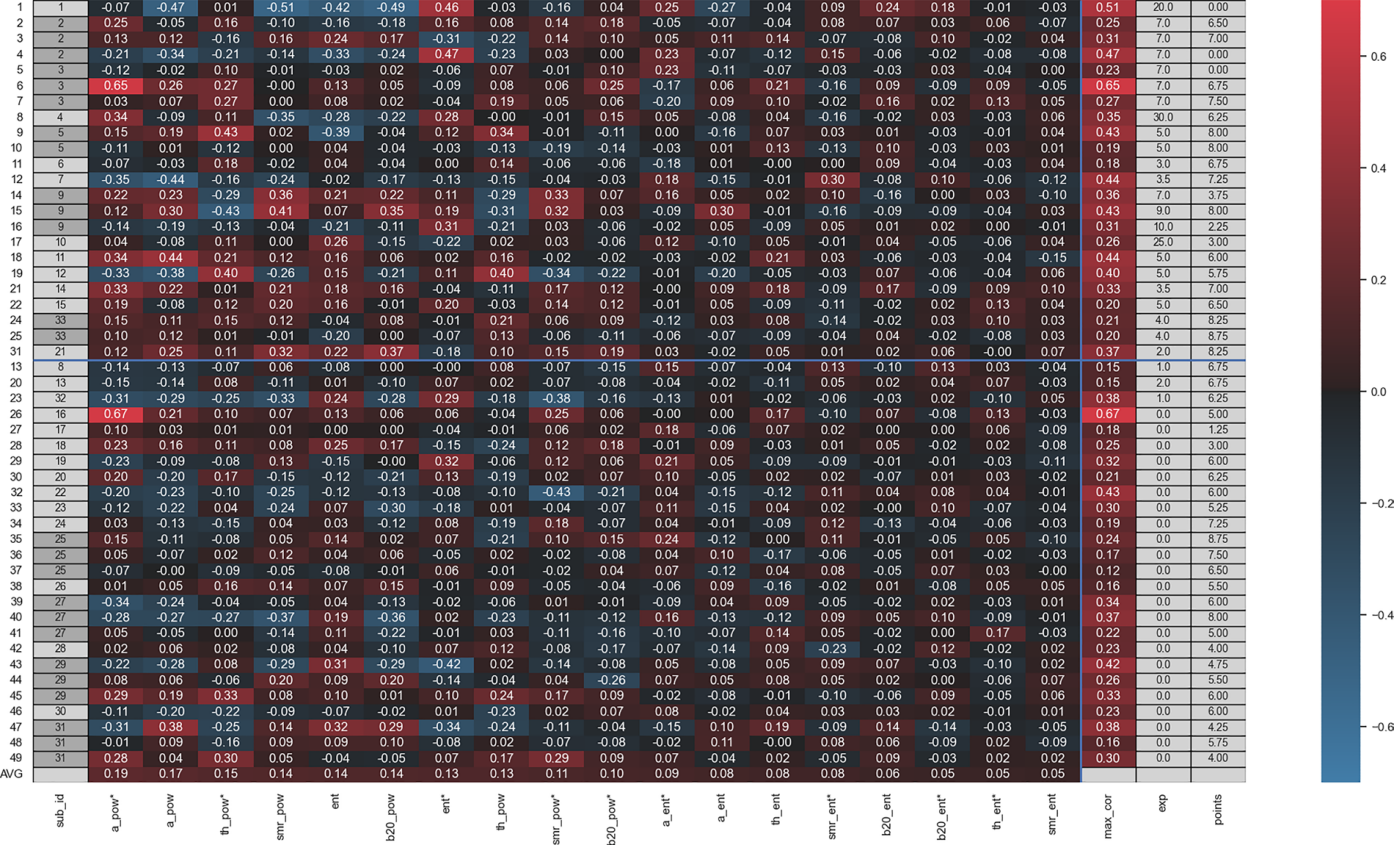
Occiptial matrix (for occiptial electrodes) that shows difference (PBCC) between concentration and mind wandering for every considered metrics. Metrics with * show difference by interquartile range (PBCCIQR). Columns are sorted based on metrics significance.

Additionally, Figs. 8–10 present similar matrices for the prefrontal, central, and occipital & parietal regions, respectively. It is evident that the differences between states are most significant in the prefrontal channels, consistent with the findings of previous studies. Although the correlation values of the metrics in the prefrontal channels are significantly higher for some individual recordings compared to the corresponding values across all derivations, the average values show no significant differences. The differences between classes within the central channels are the least significant among all those considered. For the occipital & parietal derivations, a relative increase in the significance of the power metric in the *α*-band can be observed (first column in the table). This is an expected result, given the typical spatial distribution characteristics associated with this frequency band^21^.

### ECG data validation

We have calculated 58 indexes using Kubios software based on PPI data (see Table 2). These indexes reflect the activity of the sympathetic and para-sympathetic nervous systems. We used a five minutes epochs for indexes calculation. There are three categories of indexes: time-domain, frequency-domain, and nonlinear HRV analysis methods. The first category is calculated based on beat-to-beat PPI interval values. The second category is calculated based on power spectral density for the same values. The last category includes non-linear mechanisms of heart rate regulation. One of the indices that can be used as a metric for ECG recording quality is the percentage of automatically corrected segments. In our recordings, this value does not exceed 5.5%, with an average on all experiment records being 0. 2% (0% means that any interpolations are absent, which means the best quality of the recording).

**Table 2:** HRV Parameter Descriptions.

#	Parameter (Units)	Description
**Overview**		
1	StressIndex (-)	Square root of Baevsky’s stress index
2	PNSindex (-)	Parasympathetic nervous system activity compared to normal resting values
3	SNSindex (-)	Sympathetic nervous system activity compared to normal resting values
4	HRzones (%)	Time spent in Maximum, Hard, Moderate, Light, Very light, and Inactive zones
**HR Zones**		
5	PNSzones (%)	Time spent in Very high, High, Normal, Low and Very Low PNS index levels
6	SNSzones (%)	Time spent in Very high, High, Normal, Low and Very low SNS index levels
7	Energy exp. (kcal/min)	Activity-related energy expenditure (EE) estimated using Keytel’s model
8	TRIMP (TRIMP/min)	Training impulse (TRIMP) according to Banister’s exponential model
**Time-Domain**		
9	RR (ms)	The mean of RR intervals
10	STDRR (SDNN) (ms)	Standard deviation of RR intervals
11	HR (1/min)	The mean heart rate
12	STDHR (1/min)	Standard deviation of instantaneous heart rate values
13	Min & Max HR (1/min)	Min and max HR computed using N-beat moving average (default N=5)
14	RMSSD (ms)	Root mean square of successive differences between RR intervals
15	NNxx (beats)	Number of successive RR pairs differing more than xx ms (default xx=50)
16	pNNxx (%)	NNxx divided by total number of RR intervals
17	HRV triangular index (-)	Integral of RR histogram divided by its peak height
18	TINN (ms)	Baseline width of the RR histogram
19	DC, AC (ms)	HR deceleration capacity (DC) and acceleration capacity (AC)
20	SDANN (ms)	Std. dev. of 5-minute average RR intervals
21	SDNNI (ms)	Mean of the std. dev. of RR intervals in 5-minute segments
**Frequency-Domain**		
22	Spectrum (-)	Welch’s (or Lomb-Scargle) periodogram and AR spectrum estimates
23	Peak frequency (Hz)	VLF, LF, and HF band peak frequencies
24	Absolute power (ms^2^)	Absolute powers of VLF, LF, and HF bands
25	Absolute power (log)	Natural logarithm transformed values of absolute powers of VLF, LF, and HF bands
26	Relative power (%)	Relative powers of VLF, LF, and HF bands
27	Normalized power (n.u.)	Powers of LF and HF bands in normalised units
28	LF/HF (-)	Ratio between LF and HF band powers
29	RESP (Hz)	Respiration rate (derived from ECG and RR data)
**Nonlinear**		
30	SD1 (ms)	In Poincaré plot, the standard deviation perpendicular to line-of-identity
31	SD2 (ms)	In Poincaré plot, the standard deviation along the line-of-identity
32	SD2/SD1 (-)	Ratio between SD2 and SD1
33	ApEn (-)	Approximate entropy
34	SampEn (-)	Sample entropy
35	DFA, 1 (-)	Detrended fluctuation analysis, short-term fluctuation slope
36	DFA, 2 (-)	Detrended fluctuation analysis, long-term fluctuation slope
37	D2 (-)	Correlation dimension
38	RPA (-)	Recurrence plot analysis:
39	Lmean (beats)	Mean line length
40	Lmax (beats)	Maximum line length
41	REC (%)	Recurrence rate
42	DET (%)	Determinism
43	ShanEn (-)	Shannon entropy
44	MSE (-)	Multiscale entropy for scale factor values = 1, 2, ..., 20

### Video data validation

In works^16–18^, it is shown that for recognizing such features as respiratory rate, heart rate, and blood oxygen saturation, it is sufficient to extract key points from the face and chest, which is achieved in all the published videos. Each video in the dataset was manually reviewed to ensure compliance with these requirements.

## Usage Notes

To read EEG data, the.vhdr file should be used, as it contains references to the.vmrk and.eeg files. It can be read with the MNE library using the function mne.io.read_raw_brainvision. After that, it can be processed like any other Raw object, utilizing standard MNE functions for epoching, calculating PSD, and so on. The ECG data is also included in the same Raw object and can be accessed like any other channel.

## Data Availability

The dataset is available in Hugging Face repository: https://huggingface.co/datasets/alexeykashevnik/EEGMeditation.
